# Suppression of FoxO6 by lipopolysaccharide in aged rat liver

**DOI:** 10.18632/oncotarget.6219

**Published:** 2015-10-24

**Authors:** Dae Hyun Kim, Min Hi Park, Ki Wung Chung, Min Jo Kim, Daeui Park, Bonggi Lee, Eun Kyeong Lee, Yeon Ja Choi, Nam Deuk Kim, Byung Pal Yu, Hae Young Chung

**Affiliations:** ^1^ Molecular Inflammation Research Center for Aging Intervention (MRCA), College of Pharmacy, Pusan National University, Gumjung-gu, Busan, Korea; ^2^ In silico Toxicology Research Center, Korea Institute of Toxicology, Daejeon, Korea; ^3^ Department of Physiology, The University of Texas Health Science Center at San Antonio, TX, USA

**Keywords:** FoxO6, Pak1 pathway, aging, NF-κB, Akt, Gerotarget

## Abstract

The beneficial role of FoxO during aging has been proposed for its promotion of resistance to oxidative stress and inhibition of pro-inflammatory mediators. On the other hand, NF-κB is a pro-inflammatory transcription factor which is a key mediator of inflammatory cytokine generation. However, the correlation between FoxO6 and NF-κB during aging has not fully been explored.

The main purpose of the present study was to elucidate mechanisms underlying the protective role of FoxO6 in the maintenance of cellular homeostasis under potent pro-inflammatory conditions induced by LPS. Initial experimentation revealed that reduced FoxO6 activity during aging was caused by its phosphorylation, which suppressed its transcriptional activity in aged livers. Transfection with FoxO6-wt virus and FoxO6-siRNA in HepG2 cells revealed that FoxO6 phosphorylation by LPS leads to NF-κB activation *via* Akt and Pak1 pathways. Furthermore, Pak1 activity was increased in a phosphatidylinositol 3-kinase independent manner, and LPS-induced FoxO6 phosphorylation and FoxO6 inactivation were Pak1-dependent in nuclear fractions of cells. Further revealed Pak1 phosphorylation by LPS permitted interaction between FoxO6 and Akt.

Current study suggests FoxO6 phosphorylation facilitates the nuclear translocation of NF-κB *via* Akt and Pak1 pathways induced by LPS in aged rats.

## INTRODUCTION

Many investigators have demonstrated that Forkhead transcription factor (FoxO) plays key roles in the inductions of various downstream genes involved in inhibition of cellular metabolism, cell cycle, cell death, and oxidative stress response [1]. In mammals, the FoxO family consists of the evolutionally highly conserved forkhead transcription factors, FoxO1, FoxO3, FoxO4, and FoxO6 [2], which have been shown to play important roles in the aging process [3], particularly, by suppressing the generation of reactive oxygen species (ROS). Furthermore, it has been reported FoxO family members suppress in downstream of PI3K/Akt pathways, which have been reported to be associated with insulin and oxidative stress-related diseases and the aging process [4]. In addition, FoxO activation can reduce levels of cellular oxidative stress by directly increasing the expressions of manganese superoxide dismutase (MnSOD) and catalase at the mRNA and protein levels [5]. Recently, lipopolysaccharide (LPS)-mediated ROS induced tissue damage has been reported in animal models of endotoxic shock or hepatic inflammation [6], which exhibit Akt activation leading to reduced FoxO activity.

LPS (endotoxin) is a major outer membrane component of gram-negative bacteria and is a potent inducer of hepatic inflammation. In fact, LPS is capable of stimulating inflammation, cytokine production, and the accumulation of inflammatory cells within liver [7]. Animal studies suggest the pathophysiology of endotoxic shock differs in young and aged animals, and endotoxemia is known to increase the systemic levels of inflammatory cytokines [8]. Although the effects of aging on LPS-induced inflammatory response have been well studied, little is known regarding the influence of inflammation on metabolism. Endotoxin-induced inflammation is known to affect whole-body energy metabolism, including the metabolisms of carbohydrates, lipids, and amino acids, and the disruption of energy producing metabolic processes has been implicated in endotoxin-induced organ failure [9]. On the other hand, aging is associated with altered responses to LPS-induced physiological changes, such as, inflammation and immune response [10]. In this context, it is important to note that of the many known transcription factors, redox-responsive NF-κB (nuclear factor-κB) is most vulnerable to LPS activation [11].

The key features of NF-κB are its redox responsiveness and its critical activation of many pro-inflammatory genes, including cytokines and chemokines [12, 13]. NF-κB also controls the expressions of various gene products that induce important cellular processes, such as, inflammation, cell adhesion, the cell cycle, angiogenesis, and apoptosis [14]. Under normal conditions, NF-κB resides in cytoplasm bound by its inhibitory proteins [15], and aged rodents consistently show enhanced NF-κB activity in heart, kidney, and brain tissues [14] and lead to age-related loss of tissue homeostasis [16]. These observed provide strong experimental evidence supporting the notion that the increased NF-κB activity observed in aged animals is probably due to shifts in intracellular pathways. However, it remains to be determined whether Akt activates NF-κB *via* IκB [17] or *via* phosphorylation of the catalytic p65 subunit of NF-κB [18], contributing to chronic inflammation [19].

Kenyon stated the extension of lifespan requires transcription factor FoxO [20]. In mammals, FoxO isotypes are phosphorylated and inactivated by at least two oncogenic kinases, that is, Akt and Pak1 [21, 22]. Thus, in mammals (including mice), it is probably that hyper-activation of Pak1 would shorten lifespan [23]. Furthermore, it was found healthy Pak1-deficient mice were resistant to LPS-induced degranulation (calcium release) of mast cells [23], which is the hallmark of allergic inflammatory reactions. Also, insulin-mediated activations of Akt2 and Rac1 occurs downstream of PI3K, and Rac1 stimulates actin cytoskeleton reorganization [24] and activates Pak [25] by releasing Pak from its autoinhibitory domain and allowing phosphorylation of Thr423/Thr402 of Pak1 and 2 within their activation loops [26, 27]. Pak1 is an effector protein of PI3K [28] and mediates the cellular effect of polypeptide growth factor on cell motility [29], anchorage-independent growth [30, 31], and the survival of human breast cancer cells.

Recently, Chung et al. [32] reported that FoxO6 and PGC-1α form a regulatory loop that sets the oxidative metabolism level in skeletal muscle. However, the role played by FoxO6 in aging has not been well defined. In the present study, we have investigated the inhibition of FoxO6 under LPS-induced oxidative stress in aged livers. In addition, we documented the FoxO6 phosphorylation process, and showed how both Akt and Pak1 signaling modulated FoxO6 activities induced by LPS during aging by using HepG2 cells and aged rat livers.

## RESULTS

### Modification of FoxO6 and NF-κB in LPS-treated HepG2 cells

**Figure 1:**
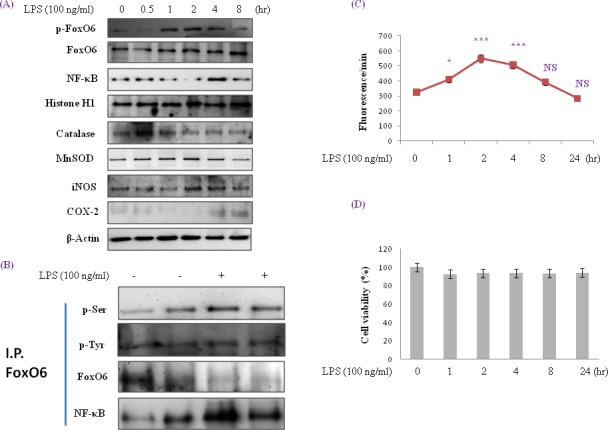
Enhancement of FoxO6 phosphorylation and NF-κB protein levels in LPS-treated HepG2 cells HepG2 cells were treated with 100 ng/ml LPS for various times. Samples loaded on sample gel were probed with β-actin and histone H1. **A.** Nuclear levels of FoxO6 and NF-κB and cytoplasmic levels of catalase, MnSOD, iNOS, and COX-2 were noticeably diminished after LPS treatment for 0.5 to 8 hr. **B.** Immunoprecipitated FoxO6 was found to be physically associated with NF-κB by Western blotting. **C.** Quantitative analysis was performed by measuring DCFDA fluorescence after treating plate with vehicle or 100 ng/ml LPS for 1 to 24h. Results were obtained using one-factor ANOVA: ***p < 0.001 *vs*. LPS untreated cells. **D.** Cells were treated with 100 ng/ml LPS for 24hr. The MTT assay used is described in Materials and Methods.

Recent evidence indicates that mammalian FoxO increases the free radical scavenger genes MnSOD and catalase, which protect against oxidative damage in human cells [33]. To investigate the role of FoxO6 in LPS-induced oxidative stress, we examined the expressions of the antioxidants catalase and MnSOD and proinflammatory genes of COX-2 and iNOS (major endogenous sources of ROS). HepG2 cells were incubated with LPS for 0.5 to 8 hr and then COX-2 and iNOS expressions were determined. As shown in Figure 1A, LPS up-regulated the expressions of these two enzymes, the expressions of which are well known to be strongly influenced by NF-κB activation. Therefore, we investigated the effect of LPS on NF-κB activity related to the expressions of iNOS and COX-2. To detect changes in p65 levels, cells were cultured for 0.5 to 8 hr in the presence of 100 ng/ml LPS. As shown in Figure 1, the incubation of cells with LPS resulted in an increase of p65 in nuclear extracts (Figure 1A). In addition, nuclear phopho-FoxO6 and NF-κB levels were noticeably increased when HepG2 cells were treated with 100 ng/ml LPS in serum-free media for 0.5 to 8 hr (Figure 1A), and COX-2 and iNOS levels were increased by LPS. These results imply that LPS increases these genes by mediating NF-κB activation.

MnSOD and catalase are two major antioxidant enzymes that play central roles in protection against oxidative stress by reducing ROS. In these experiments, protein levels of antioxidant enzymes by LPS were monitored by Western blot. As shown in Figure 1A, MnSOD levels were unchanged by LPS treatment, but catalase levels were reduced. On the other hand, p-Pak1 and p-Akt levels increased markedly after 1 hr of LPS treatment (Figure S1). We also examined the effect of FoxO6 phosphorylation on oxidative stress. As shown in Figure 1B, LPS enhanced phosphorylation of serine on FoxO6 and reduced unphosphorylated FoxO6 levels in LPS-treated HepG2 cells.

Microarray data was used to investigate the effect of LPS on gene expression in mouse livers. In the microarray data, ten genes were increased in LPS-treated livers (Figure S2). In addition, NF-κB was clearly increased in LPS treated mouse livers, whereas FoxO6 levels were suppressed (Figure S2). In addition, we found that LPS up-regulated Pak1, which has been reported to be a critical target gene of FoxO. Furthermore, both Pak1 and FoxO are regarded as components of transcriptional pathway during brain development [32].

HepG2 cells were treated with 100 ng/ml LPS in serum-free media for 1 to 24 hr (Figure 1). After 1 hr of treatment, nuclear FoxO6 levels were noticeably decreased, and its cytoplasmic levels increased markedly after 1 hr, as determined by Western blotting. In addition, we found that ROS levels were increased in an LPS-dependent manner (Figure 1C). To study the cytoprotective effects of LPS, we induced cell death by treating cells with various time of LPS. As shown in Figure 1D, cytotoxicity of LPS was not shown.

### Verification of enhanced FoxO6 phosphorylation and NF-κB up-regulation in LPS-treated HepG2 cells

**Figure 2:**
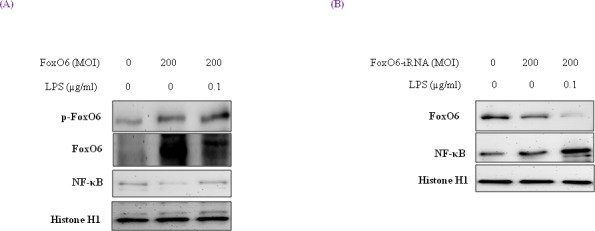
Activation of NF-κB through FoxO6 by LPS HepG2 cells were grown to 80% confluence in 100 mm dishes in DMEM, pre-treated (1 day) with or without FoxO6 (100 MOI), and then stimulated with 100 ng/ml LPS. **A.** HepG2 cells were pretransduced with 200 MOI of FoxO6 vector in the absence or presence of LPS. Cells were analyzed by Western blotting using p-FoxO6, FoxO6, NF-κB, and Histone H1 antibody. **B.** After stimulating HepG2 cells with LPS (100 ng/ml) in the absence or presence of FoxO6-siRNA (200 MOI), levels of NF-κB were determined in cell extracts.

To examine the relationship between FoxO6 and NF-κB in HepG2 cells exposed to oxidative stress, FoxO6-virus transduced cells were treated with or without LPS (100 ng/ml). As shown in Figure 2A, treatment with LPS induced remarkable shifts of nuclear phospho-FoxO6 and NF-κB, and increased NF-κB levels in FoxO6-siRNA vector-transduced cells (Figure 2B). These observations support the idea that FoxO6 suppresses proinflammatory gene up-regulation by NF-κB, and thus reducing oxidative stress.

### Effect of oxidative stress on FoxO6 phosphorylation and the PI3K/Akt pathway

Several authors have suggested that FoxOs are inhibited by the PI3K/Akt pathway. Specifically, Akt, a key downstream effector of PI3K, is thought to phosphorylate FoxOs directly [35] or to promote their phosphorylation by other kinases [35].

**Figure 3:**
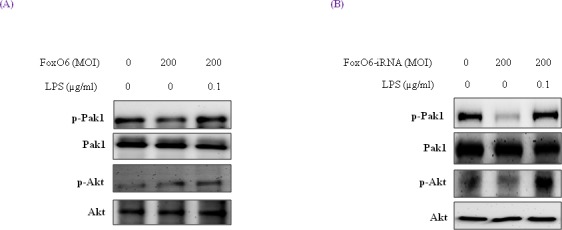
Pak1 activation and insulin signaling by LPS HepG2 cells were pre-treated (24 h) with FoxO6 (200 MOI) then stimulated with 100 ng/ml LPS. **A.** After stimulation with LPS in the absence or presence of FoxO6 (200 MOI), the phosphorylations of Pak1 and Akt were protein levels in cell extracts. One representative blot is shown from three experiments that yielded similar results. **B.** After stimulation with LPS in the absence or presence of FoxO6-siRNA (200 MOI), the phosphorylations of Pak1 and Akt were protein levels in cell extracts.

We treated HepG2 cells with FoxO6 (200 MOI) for 1 day, and then incubated them with 100 ng/ml LPS for 1 hr and assessed p-Pak1 and p-Akt levels. As shown in Figure 3A, Pak1 and Akt remained predominantly in cytosol regardless of LPS treatment or FoxO6 transduction. On the other hand, phospho-Pak1 and phospho-Akt levels were suppressed in FoxO6-siRNA transduced cells (Figure 3B).

### Downstream genes of FoxO6 and NF-κB and the involvement of the pak pathway in LPS-treated HepG2 cells

**Figure 4:**
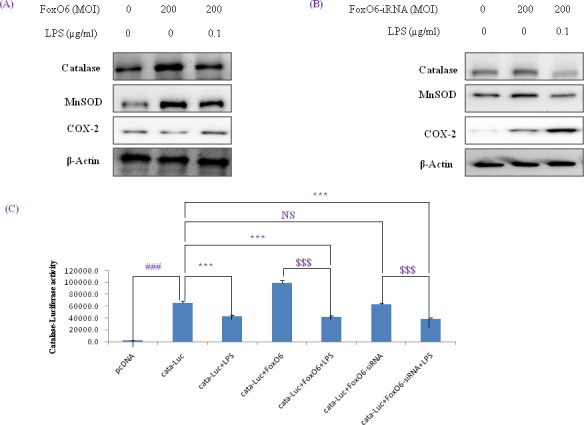
Activation of NF-κB by oxidative stress in HepG2 cells (A, B) Western blotting was used to assess COX-2, catalase, and MnSOD protein levels after pre-incubating HepG2 cells with FoxO6 (100 MOI) or FoxO6-siRNA (100 MOI) for 24 hr. **C.** Cells were transiently transfected with a catalase-containing plasmid linked to the luciferase gene, pre-incubated with FoxO6 (100 MOI) or FoxO6-siRNA (100 MOI) for 8 hr, and then treated with LPS for 2 hr. Results are presented in relative luminescence units (RLU). Results were obtained using one-factor ANOVA: ^###^*p* < 0.001 *vs*. untransduced cells; ***p < 0.001 *vs*. catalase-luciferase transduced cells; ^$$$^*p* < 0.001 *vs*. catalase-luciferase transduced cell pre-incubated with FoxO6 virus or FoxO6-siRNA.

MnSOD and catalase are two major antioxidant enzymes that act to protect cells from oxidative stress by neutralizing ROS. As shown in Figure 4A, catalase and MnSOD levels were increased by FoxO6, but reduced by LPS.

Mammalian FoxO has been shown to upregulate radical scavenger gene like catalase, which protects human cells from oxidative damage [36]. Our results showed that ROS levels were increased by LPS and decreased by FoxO6 virus transduction in HepG2 cells (data not shown).

We also examined the effect of oxidative stress on FoxO phosphorylation. As shown in Figure 1, LPS enhanced FoxO6 phosphorylation at Ser and reduced unphosphorylated FoxO6 levels in LPS-treated HepG2 cells. In addition, we treated cells with FoxO6-virus (200 MOI) for 1 day, and then incubated them with 100 ng/ml LPS for 6 hr and assessed catalase, MnSOD, and COX-2 protein levels. Catalase and MnSOD levels were noticeably increased in FoxO6-virus transduced cells and COX-2 levels were decreased (Figure 4A). In addition, catalase and MnSOD also reduced inductions levels by LPS in FoxO6-siRNA vector-transduced cells (Figure 4B).

To verify that FoxO6 binds the catalase gene for transactivation, we examined the ability of FoxO6 to stimulate catalase expression in HepG2 cells. FoxO6 was shown to be associated with catalase activity, as determined by catalase luciferase assay (Figure 4C) in FoxO6 transduced HepG2 cells. These observations support the notion that FoxO6 binds the catalase gene for transactivation, and thus, contributes to the inhibition of oxidative stress.

### Regulation of NF-κB activity by oxidative stress in HepG2 cells

**Figure 5:**
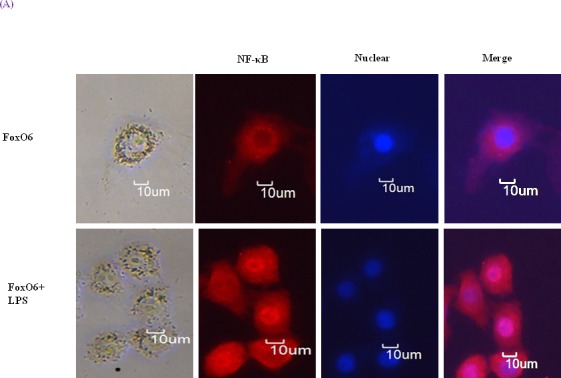

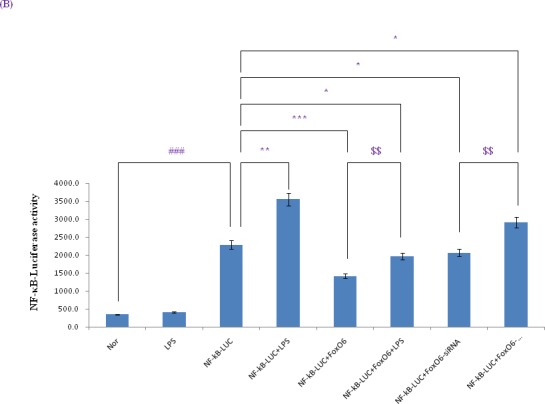

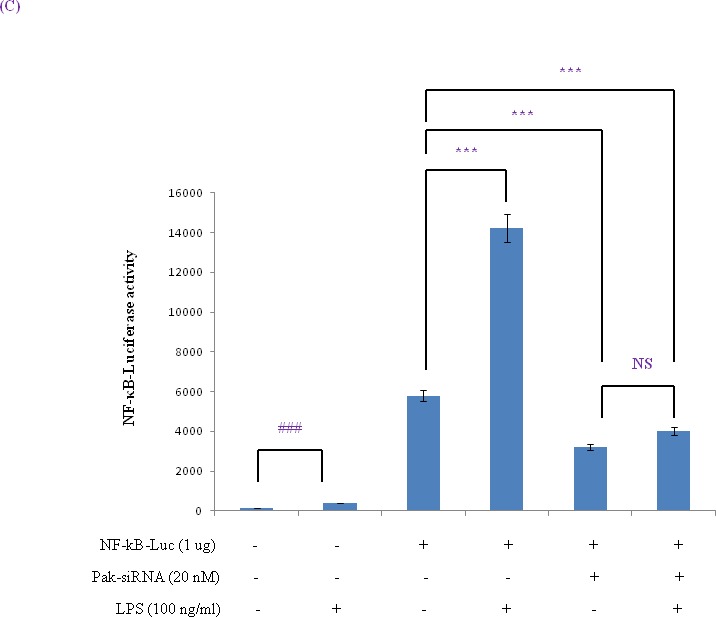
Activation of NF-κB due to FoxO6 inactivation by LPS **A.** HepG2 cells were pretransduced with 100 MOI of FoxO6 vector for 24 hr and then treated with or without LPS (100 ng/ml) for 4 hr. Cells were immunostaining using rabbit anti-NF-κB antibody followed by IgG conjugated with fluorescein isothiocyanate (Red). Bar = 10 μm. **B.** HepG2 cells were transiently transfected with a NF-κB-containing plasmid linked to the luciferase gene, pre-incubated with FoxO6 (100 MOI) or FoxO6-siRNA (100 MOI) for 24 hr and then treated with LPS for 2 hr. Results are presented in relative luminescence units (RLU). Results were obtained using one-factor ANOVA: ^###^*p* < 0.001 *vs*. untransduced cells; ^*^p < 0.05, ^**^p < 0.01, ***p < 0.001 *vs*. NF-κB-luciferase transduced cells; ^$$^*p* < 0.01 *vs*. NF-κB-luciferase transduced cells pre-incubated with FoxO6 virus or FoxO6-siRNA. **C.** HepG2 cells were transiently transfected with a NF-κB-containing plasmid linked to the luciferase gene, pre-incubated with Pak-siRNA (20 nM) for 48 hr and then treated with LPS for 2 hr. Results are presented in relative luminescence units (RLU). Results were obtained using one-factor ANOVA: ^###^*p* < 0.001 *vs*. untransduced cells; ***p < 0.001 *vs*. NF-κB-luciferase transduced cells.

To determine the extent of the age-related activation of NF-κB, EMSA was carried out on nuclear proteins. Results showed decreases in the nuclear binding activity of NF-κB in FoxO6-virus treated cells, but little change in the LPS-treated FoxO6-virus treated cells (Figure S3). NF-κB levels also increased in LPS-treated FoxO6-vector transduced cells (Figure S3). The binding specificity of NF-κB complex was investigated using a 100-fold excess of an unlabeled oligonucleotide, which competed for NF-κB binding (Figure S3, Lane 5). As shown in Figure 5A, treatment of LPS with 100 ng/ml induced a remarkable shift of NF-κB from cytoplasm to the nucleus as determined by immunostaining. To confirm that FoxO6 regulates NF-κB for transactivation, we examined the ability of FoxO6 to inhibit NF-κB activation in HepG2 cells. FoxO6 was found to be associated with NF-κB promoter, as determined by luciferase assay (Figure 5B) in FoxO6 transduced HepG2 cells. On the other hand, NF-κB activity was only slightly increased in FoxO6-siRNA treated cells (Figure 5B). We also examined the effect of NF-κB activity in the presence of Pak1 deficiency. The results obtained showed increases in NF-κB activities in LPS-treated cells, but reduced NF-κB activity in Pak1-siRNA vector-transduced cells (Figure 5C).

### Correlation between Pak1 and Akt in LPS-challenged cells

The cytokine TNF-α promotes the activation of Pak1 protein [37], and phosphorylation of Thr 423 in the active loop of Pak1 has been demonstrated to be an indicator of its activation [38, 39].

Pak1 and Akt pathways seem to act on anti-inflammatory genes *via* distinct downstream processes, a number of authors have mentioned cross talk or feedback loops between the Pak1 and Akt signaling pathways. However, FoxO is phosphorylated and inactivated by Akt and Pak1 [21, 22].

**Figure 6:**
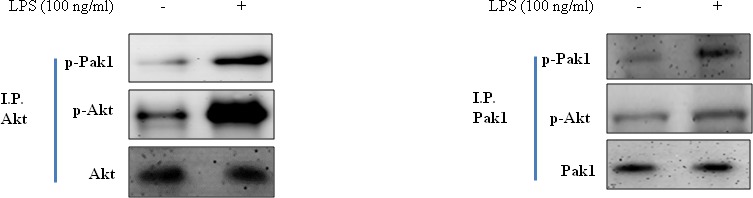
Correction between Pak1 and Akt in LPS-treated HepG2 cells Cells were treated with LPS, and immunoprecipitated Pak1 was found to be physically associated with Akt by Western blotting.

Because the Pak1 and Akt pathways play a central role in activation of stress response, we investigated inhibition of FoxO6 during aging. As shown in Figure 6, LPS enhanced Pak1 phosphorylation at Thr 423 and increased phosphorylated Akt levels in LPS-treated HepG2 cells.

These results suggest that the association between Akt and Pak1 is increased by LPS treatment.

### Effect of FoxO6 phosphorylation on the PI3K /Akt pathways in oxidative stress

**Figure 7:**
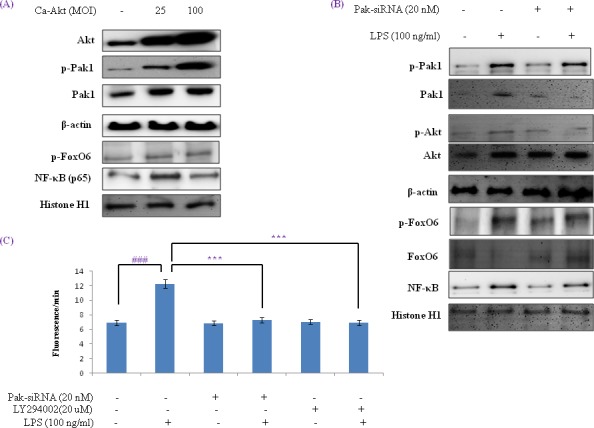
Activation of NF-κB by the Pak1 and Akt pathways in HepG2 liver cells **A.** HepG2 cells were transduced with or without Ca-Akt vector (25 or 100 MOI), and analyzed by Western blotting using Akt, p-Pak1, Pak1, β-actin, p-FoxO6, p65, and Histone H1 antibody. **B.** Cells were transfected with or not with Pak1-siRNA transfected (20 nM for 2 days) and then stimulated with 100 ng/ml LPS for 2 hr. Levels of phosphorylated Pak1, Akt, FoxO6 and NF-κB were assessed in cell extracts. **C.** Quantitative analysis was performed by measuring DCFDA fluorescence in Pak1-siRNA (20 nM, 2 days) or LY294002 (20 μM, 2h) transfected cells or non-transfected cells with vehicle or 100 ng/ml LPS for 2 hr. Results were obtained using one-factor ANOVA: ^###^*p* < 0.001 *vs*. LPS untreated cells; ***p < 0.001 *vs*. LPS treated cells.

Several authors have suggested that the FoxOs are regulated by the PI3K/Akt pathway. Specifically, Akt is believed to phosphorylate FoxOs directly [35] or to promote their phosphorylations by other kinases [35]. We examined the PI3K/Akt pathway and its effect on FoxO6 phosphorylation using constitutively active Akt (CA-Akt) and measured the phosphorylation of FoxO6 by Akt. We found that FoxO6 reduction caused by FoxO6 phosphorylation induced by an increase in Akt concentration (Figure 7A). However, phospho-Pak1 and p-Akt levels were noticeably increased after only 1 hr in CA-Akt (100 MOI) virus treated cells (Figure S4). We also examined the activations of FoxO6 and NF-κB in HepG2 cells exposed to oxidative stress in the presence of Pak1 deficiency. Cells were treated with or without LPS (100 ng/ml) in Pak1-siRNA treated cells, and as shown in Figure 7B, treatment with LPS induced the phosphorylations of FoxO6 and proinflammatory gene NF-κB. LPS also decreased Pak1 and Akt levels in Pak1-siRNA transduced cells (Figure 7B), and phospho-Pak1 and phospho-Akt levels increased markedly after 2 hr of LPS treatment (Figure S5A). In addition, we found that ROS levels were increased by LPS, but reduced in Pak-siRNA transduced groups and Akt inhibitor treated groups (Figure 7C). We examined the Pak1 and Akt pathways and their effects on FoxO6 phosphorylation utilizing IPA-3 (a Pak1 inhibitor) and LY294002 (an Akt inhibitor) and again measured the phosphorylation of FoxO6 induced by Pak1 and Akt. We found that FoxO6 phosphorylation was reduced when phospho-Pak1 and phospho-Akt levels were diminished (Figure S5B). However, phospho-Pak1 and p-Akt levels were noticeably increased after treating HepG2 cells with LPS for only 2 hr (Figure S5B). These observations support the possibility that Pak1 increases proinflammatory gene induction by NF-κB, and thus, contributes to oxidative stress (Figure 7C).

### Aging increased FoxO6 inactivity in an LPS dependent manner

It has been reported that FoxO6 mRNA is detectable in kidney, lung, and muscle tissues in fed mice, but detected low in fed liver mice [40]. To determine whether aging influences endotoxin-induced changes to FoxO6 associated with hepatic inflammation in young (6 month) and old (24 month) SD rats, we injected animals with LPS (2 mg/kg) to mimic endotoxemia and euthanized animals 12 h later.

**Figure 8:**
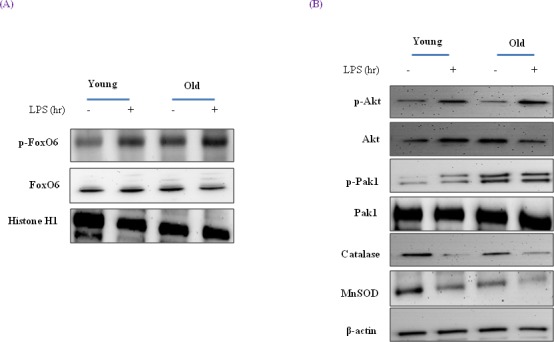
Effects of age and LPS on the expressions of FoxO6-dependent genes, MnSOD, and catalase through signaling pathways **A.** Western blot analyses for hepatic nuclear p-FoxO6 and FoxO6 were performed on nuclear proteins from LPS treated old rats. **B.** Effect of LPS treatment of old rats on the expressions of catalase, MnSOD, Pak1, and Akt. Western blot analyses were performed to determine gene and signaling protein levels in the LPS treated liver tissues of young (6 months) and old (24 months) rats. The results shown are representative of three experiments.

FoxO6 exhibited different expression patterns in young and old rats. Furthermore, phosphorylated FoxO6 levels were significantly increased in both young and old rat livers a short time after LPS injection (Figure 8A), which suggests LPS-induced inflammation in the livers of old rats was due to a decrease in FoxO6 activity.

Western blotting showed that although MnSOD and catalase levels decreased during aging (Figure 8B), catalase and MnSOD levels were greater in LPS-treated old rats than in old rats. These observations indicate the importance of FoxO6 phosphorylation during the aging process.

In addition, we examined the signaling molecules that lead to Akt activation by oxidative stress, which is known to activate PI3K and its downstream target Akt. Although Akt levels were not changed during aging (Figure 8B), LPS-treated old rats showed higher levels of phosphorylated Akt (Figure 8B). These observations support the notion that FoxO6 binds the catalase and MnSOD gene for transactivation, and thus, contributes to Akt activation.

### Effects of FoxO6 phosphorylation on age and LPS-induced inflammation

**Figure 9:**
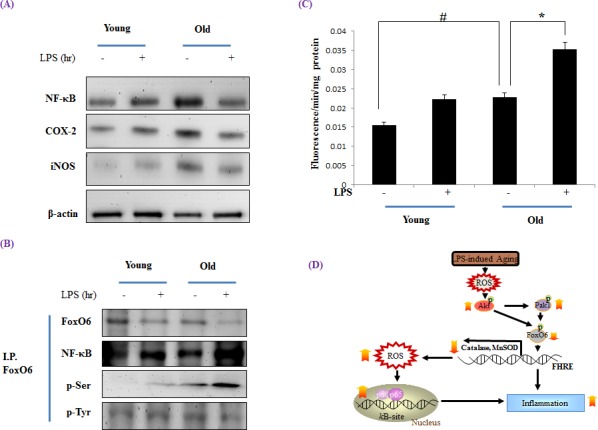
Increased NF-κB activation during LPS induced aging Western blot analyses of liver nuclear and cytosolic **A.** NF-κB, COX-2, and iNOS levels were performed using the nuclear and cytosolic proteins of LPS treated old rats. Results are representative of three independent experiments. **B.** Nuclear extracts were prepared from the liver of young and old LPS treated rats. Immunoprecipitated FoxO6 was determined to be physically associated with NF-κB by Western blotting. **C.** ROS generation in LPS treated old rats was assessed in liver homogenates using the DCFHDA method. Results are presented as means ± SEs of 4 rats. Young = 6 months old; Old = 24 months old. Results of one factor ANOVA: ^#^*p* < 0.05 vs. Young rats; ^*^p < 0.05 *vs*. LPS treated age-matched rats. **D.** Possible mechanism of the effect of FoxO6 on LPS stimulated aging. ROS, reactive oxygen species; FoxO6, Forkhead transcription factor O6; NF-κB, Nuclear transcription factor κB; Pak1, P21-activated kinase.

To determine whether NF-κB activation is increased during aging, we examined nuclear protein levels by Western blotting using p65-specific polyclonal antibodies. The nuclear translocation of NF-κB was significantly greater in old rats, but LPS-treated old rats showed higher levels of NF-κB (Figure 9A). In addition, we examined the gene expressions of iNOS and COX-2 (NF-κB-dependent genes), which possess a κB-site in their promoter regions, and found the protein levels of these two genes were increased NF-κB activity and that LPS up-regulated their expressions.

Because FoxO transcription factors play a central role in regulation of stress response [41], we investigated the modification of FoxO6 during aging. FoxO6 phosphorylation at its serine reside was increased as FoxO6 activity decreased during aging, and this was effectively counteracted by LPS-induced phosphorylation (Figure 9B). However, NF-κB also increased LPS-treated old livers.

To assess age-related oxidative status and its modulation by LPS, total ROS levels were measured in liver homogenates using a DCFDA probe. The results obtained showed that ROS levels increased with age and that this increase was significantly induced by LPS (Figure 9C). Old rat livers also exhibited LPS-induced oxidative stress, whereas young livers did not.

## DISCUSSION

Aging is associated with an increase in the inflammatory response caused by various factors [42]. LPS is a potent inducer of hepatic inflammation, and can activate inflammatory process, cytokine production, and the accumulation of inflammatory cells in liver [43, 44]. In the present study, we provide information on the molecular mechanism underlying the suppression of FoxO6 *via* LPS-induced Pak1 and Akt pathways during aging. The most significant findings of this study are decreased FoxO activity during aging is blunted by PI3K/Akt activation during aging under increased oxidative stress.

Molecular studies showed that PI3K suppressed the function of DAF-16, a Forkhead transcription factor [45], and subsequently, it was found that PI3K is critical for the proper control of metabolism and cell survival [46] and stimulates the transcription of FoxO. In mammals, FoxOs are phosphorylated and activated by AMPK [47], and that they are phosphorylated and inactivated by at least two distinct oncogenic kinases, namely, Akt and Pak1 [21, 22]. Thus, in mammals it is likely that hyper-activation of Pak1 shortens lifespan. However, activation of the PI3K/Akt-dependent mTOR and phosphorylation of FoxO1 and FoxO3 reduced MuRF-1 expression, thereby ameliorating aging-related muscle atrophy [48].

Healthy Pak1-deficient mice are resistant to the LPS-induced degranulation of mast cells [23], the hall mark of inflammatory reactions. Steckelings et al., [49] reported that LPS-induced inflammation in hyper-sensitive rats is due to the activation of NF-κB. Since Pak1 is involved in the activation of NF-κB and is essential for inflammatory response, the ability to block Pak1 and activate AMPK (a tumor suppressing kinase) simultaneously extends lifespan. Pak1 plays a pathogenic role in cancer, infectious diseases (AIDS, malaria and flu), inflammatory diseases (asthma and arthritis), insulin-resistant diabetes (type 2), hypertension, obesity, and other diseases, and thus, several synthetic drugs that selectively block Pak1 have been developed.

Pak1 is a serine/threonine protein kinase that mediates the activities of Rac1 and Cdc42 [50], and has been proposed to be an important regulator of neuronal polarity, cytoskeletal dynamics, and cell migration [28, 51]. Furthermore, Pak1 has been implicated in the cell survival pathway *via* the direct phosphorylation of cell death promoting Bad, which suggests Pak1 could neurons survive in control mice [52, 53]. PI3K stimulates Akt, which can contribute to Pak1 activation, and thus, promote cell proliferation and survival [53]. It has been reported Pak1 is stimulated by activated Akt *via* a GTPase-independent mechanism [53] and by PDK1 *via* a PI3kinase-independent mechanism [38].

NF-κB activation occurs *via* PI3K/Akt and Pak1 in a number of studies [54]. However, the targets of Pak1 in this pathway remain elusive. Pak1 has been reported to transduce signals from Ras, Raf, or Rac1 to NF-κB without activating IKKα or IKKβ [55]. However, Pak1 was identified as a direct target of FoxO in the context of controlling neuronal polarity in the mammalian brain [34]. However, the orthologous PAK-1 in *C. elegans* also acts downstream of DAF-16/FOXO during hermaphrodite-specific neurons (HSN) migration, which suggests conservation of the regulatory connection between FoxO and Pak1 during neurodevelopment [56].

Infection-induced inflammation is a major health issue for the elderly [57], and severe infections associated with aging have many risk factors [42]. However, recent studies have demonstrated innate immune responses and cytokine production in response to infection differ in the young and elderly [8, 10]. Although many predisposing factors link mortality and aging, increased inflammation may well be associated with increased morbidity and mortality [8, 10, 42].

Our study shows FoxO6 was inhibited by an increase in inflammatory response to LPS in old rat livers *via* the Pak1 and Akt pathways. Furthermore, when liver cells were exposed to inflammatory LPS, the PI3K/Akt pathways were activated, FoxO6 was phosphorylated at Ser 184, and downstream gene expressions were inhibited (Figure 4). In addition, enhanced phospho-FoxO6 reduced catalase and MnSOD levels, increased NF-κB, and induced inflammation in LPS-treated old rat livers. In HepG2 cells, LPS enhanced Pak1 activity and this was inhibited by Pak1-siRNA, which increased LPS-induced NF-κB activation by inactivating FoxO6.

Our findings provide molecular insight of the transcriptional activity of FoxO6 and reveal increased phosphorylation of PI3K/Akt during aging, which may be age-associated with elevated levels of oxidative stress. Based on our *in vivo* and *in vitro* observations, we propose the age-related phosphorylation of FoxO6 represses the expression of catalase, and that the transcription factors FoxO6 are suppressed by the activations of Pak1 and Akt when age-related oxidative stress is augmented by LPS (Figure 9D).

## MATERIALS AND METHODS

### Animals

Specific pathogen-free male Fischer 344 rats (6 or 24 months old) were obtained from Samtako (Osan, Korea) and fed a diet of the following composition: 21% soybean protein, 15% sucrose, 43.65% dextrin, 10% corn oil, 0.15% α-methionine, 0.2% choline chloride, 5% salt mix, 2% vitamin mix, and 3% Solka-Floc fiber. The *ad libitum* fed animals had free access to food, beginning at 6 weeks of age. To investigate the effects of inflammation on the aging process, young (6 months) and old (12 months) rats were injected intraperitoneally (i.p.) with LPS (2 mg/kg body weight). 12 hours later, animals were sacrificed by decapitation and livers were quickly removed, rinsed in iced-cold buffer [100 mM Tris, 1 mM EDTA, 0.2 mM phenylmethyl-sulfonylfluoride (PMSF), 1 μM pepstatin, 2 μM sodium orthovanadate (pH 7.4)], immediately frozen in liquid nitrogen, and stored at −80°C. Livers were selected for the study because they are metabolically active and sensitive to many age-related changes, such as, redox responsive molecular events. In addition, our lab has substantial experience and data on age-related liver changes.

The animal protocol used in this study was reviewed and approved beforehand by the Pusan National University-Institutional Animal Care and Use Committee (PNU-IACUC) with respect to ethicality and scientific care.

### Cell culture system

HepG2 cells (human hepatocellular carcinoma) were obtained from the ATCC (American Type Culture Collection, Rockville, MD, USA). Cells were cultured in Dulbecco's Modified Eagle Medium (DMEM) (Nissui Co., Tokyo) supplemented with 10% heat-inactivated (56°C for 30 min) fetal bovine serum (Gibco, Grand Island, NY), 233.6 mg/mL glutamine, 100 mg/mL penicillin streptomycin, and 0.25 μg/mL amphotericin B. Cells were maintained at 37°C in a humidified 5% CO_2_/95% air atmosphere.

### Materials

All chemical reagents were obtained from Sigma (St. Louis, MO, USA), except where noted. 2′,7′-Dichlorodihydrofluorescein was from Molecular Probes, Inc. (Eugene, OR, USA). Western blotting detection reagents were purchased from Amersham (Bucks, UK). RNAzol^TM^ B was obtained from TEL-TEST, Inc. (Friendwood, TX, USA). Antibodies against catalase, MnSOD, β-actin, Histone H1, p-ser, p-tyr, p-Akt, total-Akt, NF-kB, p-Pak1, total-Pak1, iNOS, and COX-2 were obtained from Santa Cruz Biotechnology (Santa Cruz, CA, USA). Antibodies against FoxO6, p-FoxO6 (Ser184) were obtained from Dr. H. H. Dong (University of Pittsburgh, PA). Anti-rabbit IgG-horseradish peroxidase-conjugated antibody and anti-mouse IgG-horseradish peroxidase-conjugated antibody were from Amersham (Bucks, UK). Horseradish peroxidase-conjugated donkey anti-sheep/goat IgG was purchased from Serotec (Oxford, UK). Polyvinylidene difluoride (PVDF) membranes were obtained from the Millipore Corporation (Bedford, MA, USA).

### Nuclear extract preparation

Frozen rat liver tissues (0.2-0.4 μg) were rinsed in PBS buffer and then transferred to a Dounce tissue grinder (Wheaton Manufacturers, NJ). Solution A (10 mM HEPES pH 7.9, 10 mM KCl, 0.1 mM EDTA, 0.1 mM EGTA, 1 mM DTT, 0.5 mM PMSF) was then added to tissues (2.5 g/ml). Tissues were homogenized with five strokes of a pestle and after adding NP-40 (0.5%) further homogenized with an additional five strokes. Homogenates were transferred to Eppendorf tubes and centrifuged in a microcentrifuge (Beckman) for 5 min at 12000 rpm.

Supernatants contained predominantly cytoplasmic constituents. To obtain nuclear pellets, 400 μl of solution C (20 mM HEPES pH 7.9, 0.4 M NaCl, 1 mM of each of EDTA, EGTA, DTT and PMSF) was added to supernatants. Tubes were mixed thoroughly and placed on a small rotatory shaker for 15 min. Finally, they were centrifuged at 12000 rpm for 10 min in a microcentrifuge. Supernatants, which contained nuclear proteins, were then removed, transferred carefully to fresh tubes, and stored at −80°C until required for Western blotting. Protein contents were determined using the Bicinchoninic Acid Protein Assay (Sigma).

### Western blotting

Western blotting was carried out as described previously [58]. Homogenized samples were boiled for 5 min with gel-loading buffer (125 mM Tris-Cl, 4% SDS, 10% 2-mercaptoethanol, pH 6.8, 0.2% bromophenol blue) at a ratio of 1:1. Total protein-equivalents of samples were separated by SDS-PAGE using acrylamide gels as described by Laemmli [59] and then transferred to PVDF membranes at 15 V for 1 hr using a semi-dry transfer system. Membranes were then immediately placed into a blocking buffer 10 mM Tris (pH 7.5), 100 mM NaCl, and 0.1% Tween-20 containing 1% non-fat milk. Blots were allowed to block at room temperature for 1 hr, and membranes were incubated with the appropriate specific primary antibody at 25°C for 1 hr, followed by horseradish peroxidase-conjugated secondary antibody at 25°C for 1 hr. Antibody labeling was detected using enhanced chemiluminescence, according to the manufacturer's instructions. Molecular weights were determined using pre-stained protein markers.

### Transfection and luciferase reporter assay

Catalase activities were assessed using Catalase-Luc vector (Dr. Dong, University of Pittsburgh, PA, USA) containing a specific binding sequence for FoxO6. Transfection was carried out using Lipofectamine 2000 (Invitrogen). Briefly, 1 × 10^4^ cells per well were seeded in 48-well plates, cultured until ∼40% confluent, and treated with 1 μg DNA/0.5 μl Lipofectamine 2000 complexes in 500 μl of media containing 10% serum for 24 hr. HepG2 cells were treated with FoxO6 (100 MOI) virus or FoxO6-siRNA (100 MOI) 24 hr after transfection. Cells were then treated with 100 μM of LPS for 2 hr, washed with PBS, and assayed using the Steady-Glo Luciferase Assay System (Promega, Madison, WI, USA). Luciferase activities were measured using a luminometer (GENious, TECAN, Salzburg, Austria).

### Reactive oxygen species (ROS) scavenging activity assay

ROS generation in tissues was quantified as previously described [58]. DCFDA (125 μM; 2′, 7′-dichlorodihydrofluorescein diacetate) was added to 10 μl homogenate with buffer to a final volume of 250 μl.

To assess intracellular ROS activities, HepG2 cells were seeded in a 96-well plate and one day later, the medium was changed to fresh, serum-free medium. Cells were treated with or without FoxO6-virus, FoxO6-siRNA, and/or CA-Akt virus and pre-incubated for 1 day. After treatment with LPS (100 μg/ml) for 2h, media were replaced with fresh, serum free medium, and DCFDA (2.5 μM) was added. DCF fluorescence intensities measured every 5 min for 1 hr using a microplate fluorescence reader TECAN (Salzburg, Austria) and excitation and emission wavelengths of 485 and 535 nm, respectively.

### Immunoprecipitation (IP) of nuclear extracts

Nuclear extracts were immunoprecipitated in a buffer containing 40 mM Tris-HCl (pH 7.6), 120 mM NaCl, 20 mM β-glycerophosphate, 20 mM NaF, 2 mM sodium orthovanadate, 5 mM EDTA, 1 mM PMSF, 0.1% NP40, leupeptin (2 μg/ml), aprotinin (1 μg/ml), and pepstatin A (1 μg/ml) [58]. Aliquots of nuclear extracts were than precleared using a 50% protein A agarose for 30 min at 4°C, centrifuged at 12,000 g at 4°C for 15 min, incubated overnight at 4°C with the required antibody, and then incubated overnight at 4°C with 50% protein A agarose slurry. After washing immunoprecipitates three times with IP buffer, immunoprecipitated proteins were analyzed by SDS-PAGE, and Western blotting analysis was performed as described above.

### Immunostaining

HepG2 cells were seeded at 1×10^4^ cells per well in a 12-well plate, incubated for 24 hr, fixed in 4% paraformaldehyde solution (15 min at room temperature), washed with PBS buffer, blocked with 3% normal goat serum (Gibco, Grand Island, USA), and immunostained with rabbit anti-NF-κB antibody (1:1000 dilution; Santa Cruz, CA) at 4°C overnight. Cells were then washed with TBS and incubated for 3 h in the presence of anti-rabbit IgG labeled with Alexa Fluor 488 (1:200; Invitrogen, CA, USA). Cell nuclei were visualized by immunostaining with Hoechst 33342 (1:1000; Invitrogen), and NF-κB antibody were determined by confocal laser scanning microscopy (TCS SP2, Leica, Wetzler, Germany).

### Electrophoretic mobility shift (EMSA)

EMSA was used to study NF-κB binding to DNA [60]. NF-κB protein was prepared from HepG2 cells pretransduced with FoxO6 vector at an MOI of 100 pfu/cell. Oligonucleotides were labeled with biotin using the Biotin 3′-End DNA Labeling kit (Pierce Biotechnology) self-annealed to form double-stranded biotin-labeled DNA. EMSA was performed using the Lightshift Chemiluminescent EMSA kit (Pierce Biotechnology). For DNA supershift assays, aliquots of anti-FoxO6 antibody (1 μg, rabbit anti-FoxO6) were included in the EMSA assay.

### Microarray analysis of the LPS rat model

To identify intermediate molecules between FoxO6 and NF-κB, we used microarrays of LPS-treated rat liver (GSE38078) obtained from the Gene Expression Omnibus database (http://www.ncbi.nlm.nih.gov/geo/). Microarrays were produced from eight Sprague-Dawley rats which had received either an intraperitoneal injection of LPS (3 mg/kg body weight; *N* = 4) or sodium chloride (SC) 0.9% (*N* = 4) [61]. We selected 20 inflammation-related genes and genes involving FoxO, such as, Akt, FoxO, iNOS, and NF-κB. Gene expressions were reanalyzed by normalization and hierarchical clustering using the MeV program [62].

### Statistical analysis

ANOVA was conducted to determine the significances of differences between groups. Fisher's Protected LSD post hoc test was used to determine the significances of differences between group means. Statistical significance was accepted for p values < 0.05.

## SUPPLEMENTARY MATERIAL FIGURES



## Abbreviations

FoxO6: Forkhead box O6
PKB: Protein kinase B
Daf-16: Abnormal Dauer formation 16
ChIP: Chromatin immunoprecipitation
Pak1: p21-activated kinase
NF-κB: Nuclear transcription factor Κb
PI3K: Phosphoinositide 3-kinase

## References

[1] Accili D, Arden KC (2004). FoxOs at the crossroads of cellular metabolism, differentiation, and transformation. Cell.

[2] Van der Heide LP, Hoekman MFM, Smidt MP (2004). The ins and outs of FoxO shuttling: mechanisms of FoxO translocation and transcriptional regulation. Biochem J.

[3] Chung HY, Lee EK, Choi YJ, Kim JM, Kim DH, Zou Y, Kim CH, Lee J, Kim HS, Kim ND, Jung JH, Yu BP (2011). Molecular Inflammation as an Underlying Mechanism of the Aging Process and Age-related Diseases. J Dent Res.

[4] Karger S, Weidinger C, Krause K, Sheu SY, Aigner T, Gimm O, Schmid KW, Dralle H, Fuhrer D (2009). FOXO3a: a novel player in thyroid carcinogenesis?. Endocr Relat Cancer.

[5] Burgering BM, Medema RH (2003). Decisions on life and death: FOXO Forkhead transcription factors are in command when PKB/Akt is off duty. J Leukoc Biol.

[6] Foo NP, Lin SH, Lee YH, Wu MJ, Wang YJ (2011). Alpha-lipolic acid inhibits liver fibrosis through the attenuation of ROS-triggered signaling in hepatic stellate cells activated by PDGF and TGF-beta. Toxicology.

[7] Affo S, Morales-Ibanez O, Rodrigo-Torres D, Altamirano J, Blaya D, Dapito DH, Millan C, Coll M, Caviglia JM, Arroyo V, Caballeria J, Schwabe RF, Gines P (2014). CCL20 mediates lipopolysaccharide induced liver injury and is a potential driver of inflammation and fibrosis in alcoholic hepatitis. Gut.

[8] Turnbull IR, Clark AT, Stromberg PE, Dixon DJ, Woolsey CA, Davis CG, Hotchkiss RS, Buchman TG, Coopersmith CM (2009). Effects of aging on the immunopathologic response to sepsis. Crit Care Med.

[9] Carre JE, Singer M (2008). Cellular energetic metabolism in sepsis: the need for a systems approach. Biochim Biophys Acta.

[10] Saito H, Sherwood ER, Varma TK, Evers BM (2003). Effects of aging on mortality, hypothermia, and cytokine induction in mice with endotoxemia or sepsis. Mech Ageing Dev.

[11] Fujihara M, Muroi M, Tanamoto K, Suzuki T, Azuma H, Ikeda H (2003). Molecular mechanisms of macrophage activation and deactivation by lipopolysaccharide: role of the receptor complex. Pharmacol Ther.

[12] Sung B, Park S, Yu BP, Chung HY (2004). Modulation of PPAR in aging, inflammation, and calorie restriction. J Gerontol A Biol Sci Med Sci.

[13] Rahman I, Marwick J, Kirkham P (2004). Redox modulation of chromatin remodeling: impact on histone acetylation and deacetylation, NF-kappaB and pro-inflammatory gene expression. Biochem Pharmacol.

[14] Kim HJ, Jung KJ, Yu BP, Cho CG, Chung HY (2002). Influence of aging and calorie restriction on MAPKs activity in rat kidney. Exp Gerontol.

[15] Baldwin AS (1996). The NF-κB and IκB proteins: new discoveries and insights. Annu Rev Immunol.

[16] Osorio FG, López-Otín C, Freije JM (2012). NF-kB in premature aging. Aging (Albany NY).

[17] Ozes ON, Mayo LD, Gustin JA, Pfeffer SR, Pfeffer LM, Donner DB (1999). NF-kappaB activation by tumour necrosis factor requires the Akt serine-threonine kinase. Nature.

[18] Sizemore N, Leung S, Stark GR (1999). Activation of phosphatidylinositol 3-kinase in response to interleukin-1 leads to phosphorylation and activation of the NF-kappaB p65/RelA subunit. Mol Cell Biol.

[19] Bektas A, Zhang Y, Lehmann E, Wood WH 3rd, Becker KG, Madara K, Ferrucci L, Sen R (2014). Age-associated changes in basal NF-κB function in human CD4+ T lymphocytes via dysregulation of PI3 kinase. Aging (Albany NY).

[20] Kenyon C (2010). A pathway that links reproductive status to lifespan in C. eleganas. Ann N Y Acad Sci.

[21] Mazumdar A, Kumar R (2003). Estrogen regulation of Pak1 and FKHR pathways in breast cancer cells. FEBS Letters.

[22] Plas DR, Thompson CB (2003). Akt activation promotes degradation of tuberin and FOXO3a *via* the proteasome. J Biol Chem.

[23] Allen JD, Jaffer ZM, Park SJ, Burgin S, Hofmann C, Sells MA, Chen S, Derr-Yellin E, Michels EG, McDaniel A, Bessler WK, Ingram DA, Atkinson SJ (2009). p21-activated kinase regulates mast cell degranulation *via* effects on calcium mobilization and cytoskeletal dynamics. Blood.

[24] Tapon N, Hall A (1997). Rho, Rac and Cdc42 GTPases regulate the organization of the actin cytoskeleton. Curr Opin Cell Biol.

[25] Manser E, Leung T, Salihuddin H, Zhao ZS, Lim L (1994). A brain serine/threonine protein kinase activated by cdc42 and Rac1. Nature.

[26] Sylow L, Jensen TE, Kleinert M, Hojlund K, Kiens B, Wojtaszewski JF, Prats C, Schjerling P, Richter EA (2013). Rac1 signaling is required for insulin-stimulated glucose uptake and is dysregulated in insulin-resistance murine and human skeletal muscle. Diabetes.

[27] Zhao ZS, Manser E, Chen XQ, Chong C, Leung T, Lim L (1998). A conserved negative regulatory region in aPAK: Inhibition of PAK kinases reveals their morphological roles downstream of cdc42 and Rac1. Mol Cell Biol.

[28] Sells MA, Knaus UG, Bagrodia S, Ambrose DM, Bokoch GM, Chernoff J (1997). Human p21-activated kinase (Pak1) regulates actin organization in mammalian cells. Curr Biol.

[29] Adam L, Vadlamudi R, Kondapaka SB, Chernoff J, Mendelsohn J, Kumar R (1998). Heregulin regulates cytoskeletal reorganization and cell migration through the p21-activated kinase-1 *via* phosphatidylinositol-3 kinase. J Biol Chem.

[30] Vadlamudi RK, Adam L, Wang RA, Mandal M, Nguyen D, Sahin A, Chernoff J, Hung MC, Kumar R (2000). Regulatable expression of p21-activated kinase-1 promotes anchorage-independent growth and abnormal organization of mitotic spindles in human epithelial breast cancer cells. J Biol Chem.

[31] Howe AK, Juliano RL (2000). Regulation of anchorage-dependent signal transduction by protein kinase A and p21-activated kinase. Nat Cell Biol.

[32] Chung SY, Huang WC, Su CW, Lee KW, Chi HC, Lin CT, Chen ST, Huang KM, Tsai MS, Yu HP, Chen SL (2013). FoxO6 and PGC-1a form a regulatory loop in myogenic cells. Biosci Rep.

[33] Kops GJ, Dansn TB, Polderman PE, Saarloos I, Wirtz KW, Coffer PJ, Huang TT, Bos JL, Medema RH, Burgering BM (2002). Forkhead transcription factor FOXO3a protects quiescent cells from oxidative stress. Nature.

[34] de la Torre-Ubieta L, Gaudillière B, Yang Y, Ikeuchi Y, Yamada T, DiBacco S, Stegmüller J, Schüller U, Salih DA, Rowitch D, Brunet A, Bonni A (2010). A FOXO-Pak1 transcriptional pathway controls neuronal polarity. Genes Dev.

[35] Brunet A, Bonni A, Zigmond MJ, Lin MZ, Juo P, Hu LS, Anderson MJ, Arden KC, Blenis J, Greenberg ME (1999). Akt promotes cell survival by phosphorylating and inhibiting a Forkhead transcription factor. Cell.

[36] Kerr LD (1995). Electrophoretic mobility shift assay. Methods Enzymol.

[37] Hsu HY, Twu YC (2000). Tumor necrosis factor-alpha-mediated protein kinases in regulation of scavenger receptor and foam cell formation on macrophage. J Biol Chem.

[38] King CC, Gardiner EMM, Zenke FT, Bohl BP, Newton AC, Hemming BA, Bokoch GM (2000). p21-activated kinase (PAK1) is phosphorylated, and activated by 3-phosphoinisitide-dependent kinase-1 (PDK1). J Biol Chem.

[39] Zenke FT, King CC, Bohl BP, Bokoch GM (1999). Identification of a central phosphorylation site in p21-activated kinase regulating autoinhibition and kinase activity. J Biol Chem.

[40] Kim DH, Perdomo G, Zhang T, Slusher S, Lee S, Phillips BE, Fan Y, Giannoukakis N, Gramignoli R, Strom S, Ringquist S, Dong HH (2011). FoxO6 integrates insulin signaling with gluconeogenesis in the liver. Diabetes.

[41] Kim HS, Skurk C, Maatz H, Shiojima I, Ivashchenko Y, Yoon SW, Park YB, Walsh K (2005). Akt/FoxO3a signaling modulates the endothelial stress response through regulation of heat shock protein 70 expression. FASEB J.

[42] Opal SM, Girard TD, Ely EW (2005). The immunopathogenesis of sepsis in elderly patients. Clin Infect Dis.

[43] Miele L, Valenza V, La Torre G, Montalto M, Cammarota G, Ricci R, Masciana R, Forgione A, Gabrieli ML, Perotti G, Vecchio FM, Rapaccini G, Gasbarrini G (2009). Increased intestinal permeability and tight junction alterations in nonalcoholic fatty liver disease. Hepatology.

[44] Chen X, Zhang C, Zhao M, Shi CE, Zhu RM, Wang H, Zhao H, Wei W, Li JB, Xu DX (2011). Melatonin alleviates lipopolysaccharide-induced hepatic SREBP-1c activation and lipid accumulation in mice. J Pineal Res.

[45] Lin K, Dorman JB, Rodan A, Kenyon C (1997). daf-16: An HNF-3/forkhead family member that can function to double the life-span of Caenorhabditis elegans. Science.

[46] Birkenkamp KU, Coffer PJ (2003). Regulation of cell survival and proliferation by the FOXO (Forkhead box, class O) subfamily of Forkhead transcription factors. Biochem Soc Trans.

[47] Greer EL, Oskoui PR, Banko MR, Maniar JM, Gygi MP, Gygi SP, Brunet A (2007). The energy sensor AMP-activated protein kinase directly regulates the mammalian FOXO3 transcription factor. J Biol Chem.

[48] Kimura K, Cheng XW, Inoue A, Hu L, Koike T, Kuzuya M (2014). β-Hydroxy-β-methylbutyrate facilitates PI3K/Akt-dependent mammalian target of rapamycin and FoxO1/3a phosphorylations and alleviates tumor necrosis factor α/interferon γ-induced MuRF-1 expression in C2C12 cells. Nutr Res.

[49] Steckelings UM, Larhed M, Hallberg A, Widdop RE, Jones ES, Wallinder C, Namsolleck P, Dahlöf B, Unger T (2011). Non-peptide AT2-receptor agonists. Curr Opin Pharmacol.

[50] Bokoch GM (2003). Biology of the p21-activated kinases. Annu Rev Biochem.

[51] Jacobs T, Causeret F, Nishimura YV, Terao M, Norman A, Hoshino M, Nikolic M (2007). Localized activation of p21-activated kinase controls neuronal polarity and morphology. J Neurosci.

[52] Schurmann A, Mooney AF, Sanders LC, Sells MA, Wang HG, Reed JC, Bokoch GM (2000). p21-activated kinase 1 phosphorylates the death agonist bad and protects cells from apoptosis. Mol Cell Biol.

[53] Tang Y, Zhou H, Chen A, Pittman RN, Field J (2000). The Akt proto-oncogene links Ras to Pak and cell survival signals. J Biol Chem.

[54] Frost JA, Swantek JL, Stippec S, Yin MJ, Gaynor R, Cobb MH (2000). Stimulation of NF-kB activity by multiple signaling pathways requires PAK1. J Biol Chem.

[55] Dadke D, Fryer BH, Golemis EA, Field J (2003). Activation of p21-activated kinase 1-nuclear factor kappaB signaling by Kaposi's sarcoma-associated herpes virus G protein-coupled receptor during cellular transformation. Cancer Res.

[56] Kennedy LM, Pham SC, Grishok A (2013). Nonautonomous regulation of neuronal migration by insulin signaling, DAF-16/FOXO, and PAK-1. Cell Rep.

[57] Martin GS, Mannino DM, Moss M (2006). The effect of age on the development and outcome of adult sepsis. Crit Care Med.

[58] Kim DH, Kim JY, Yu BP, Chung HY (2008). The activation of NF-kappaB through Akt-induced FOXO1 phosphorylation during aging and its modulation by calorie restriction. Biogerontology.

[59] Laemmli UK (1970). Cleavage of structural proteins during the assembly of the head of bacteriophage T4. Nature.

[60] Kim DH, Park MH, Choi YJ, Chung KW, Park CH, Jang EJ, An HJ, Yu BP, Chung HY (2013). Molecular study of dietary heptadecane for the anti-inflammatory modulation of NF-kB in the aged kidney. PLoS One.

[61] Wang Q, Somwar R, Bilan PJ, Liu Z, Jin J, Woodgett JR, Klip A (1999). Protein kinase B/Akt participates in GLUT4 translocation by insulin in L6 myoblasts. Mol Cell Biol.

[62] Saeed AI, Sharov V, White J, Li J, Liang W, Bhagabati N, Braisted J, Klapa M, Currier T, Thiagarajan M, Sturn A, Snuffin M, Rezantsev A (2003). TM4: a free, open-source system for microarray data management and analysis. Biotechniques.

