# Supplementary material for: Suppression of FoxO6 by lipopolysaccharide in aged rat liver
Source: Oncotarget. 2015 Oct 24;6(33):34143–57. doi: 10.18632/oncotarget.6219 (PMC4741442; doi:10.18632/oncotarget.6219)
Supplement: Supplementary file 1 [file oncotarget-06-34143-s001.pdf]

Suppression of FoxO6 by lipopolysaccharide in aged rat liver

Supplementary Material

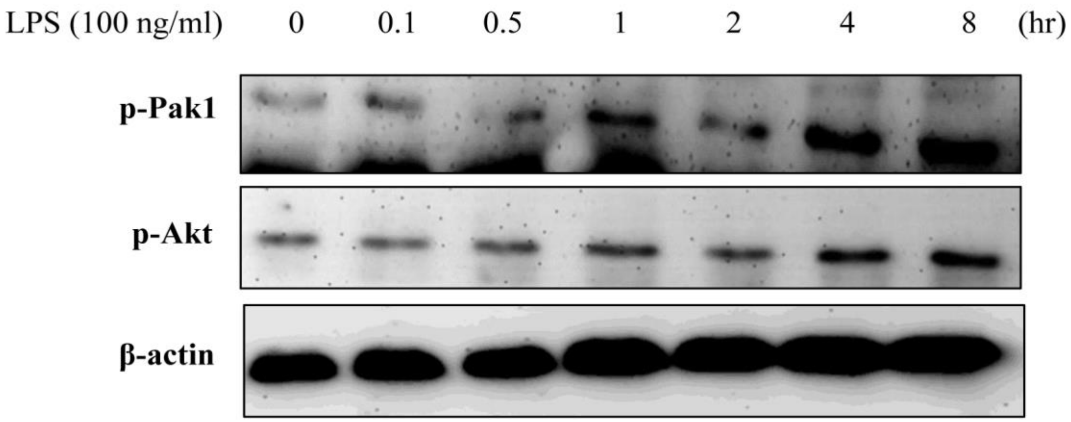

Figure S1. Enhancements of the phosphorylations of Pak1 and Akt in LPS-treated HepG2 cells. Pak1 and Akt activation levels were determined by Western blotting after treating HepG2 cells with 100 ng/ml LPS for various times. Samples loaded on sample gel were probed with β-actin. phosphorylated cytoplasmic Pak1 and Akt levels were noticeably diminished by LPS treatment at 0.1 to 8 hr.

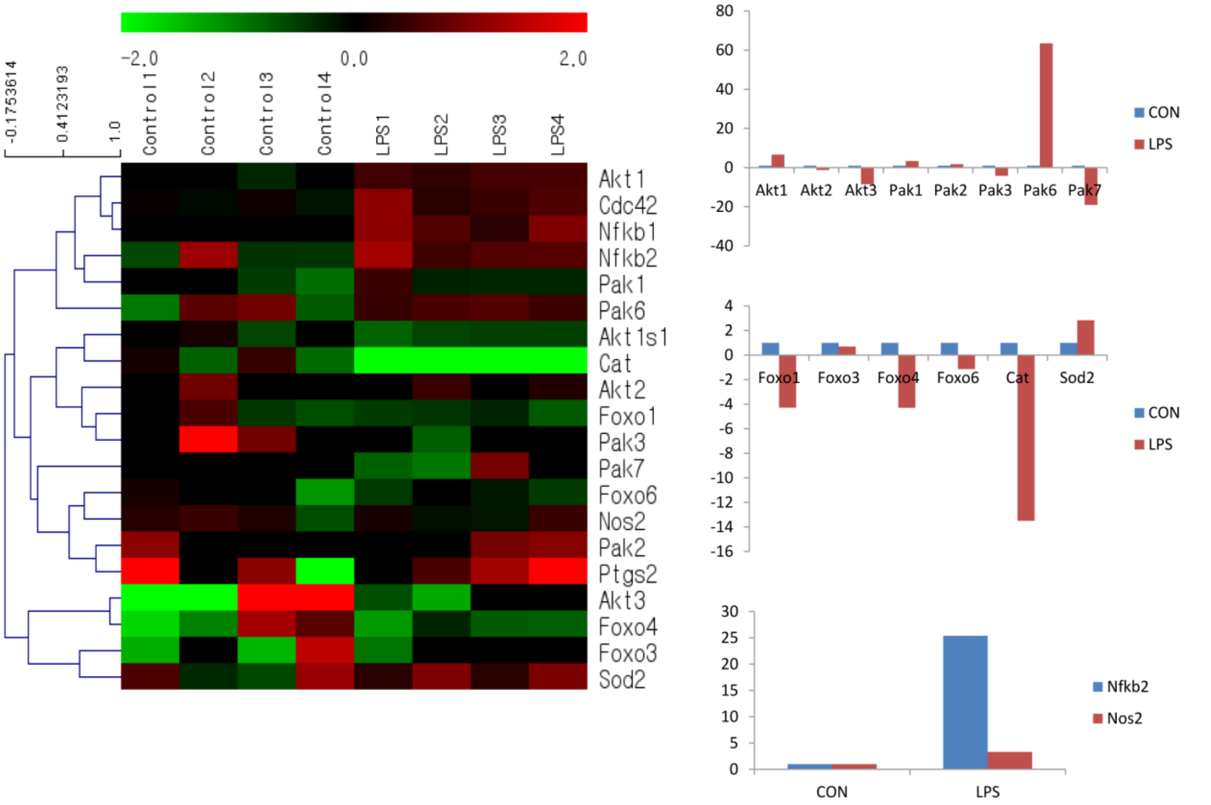

Figure S2. Microarray analysis of LPS treated mouse livers. This liver map shows the ten genes expressionally altered by LPS treatment.

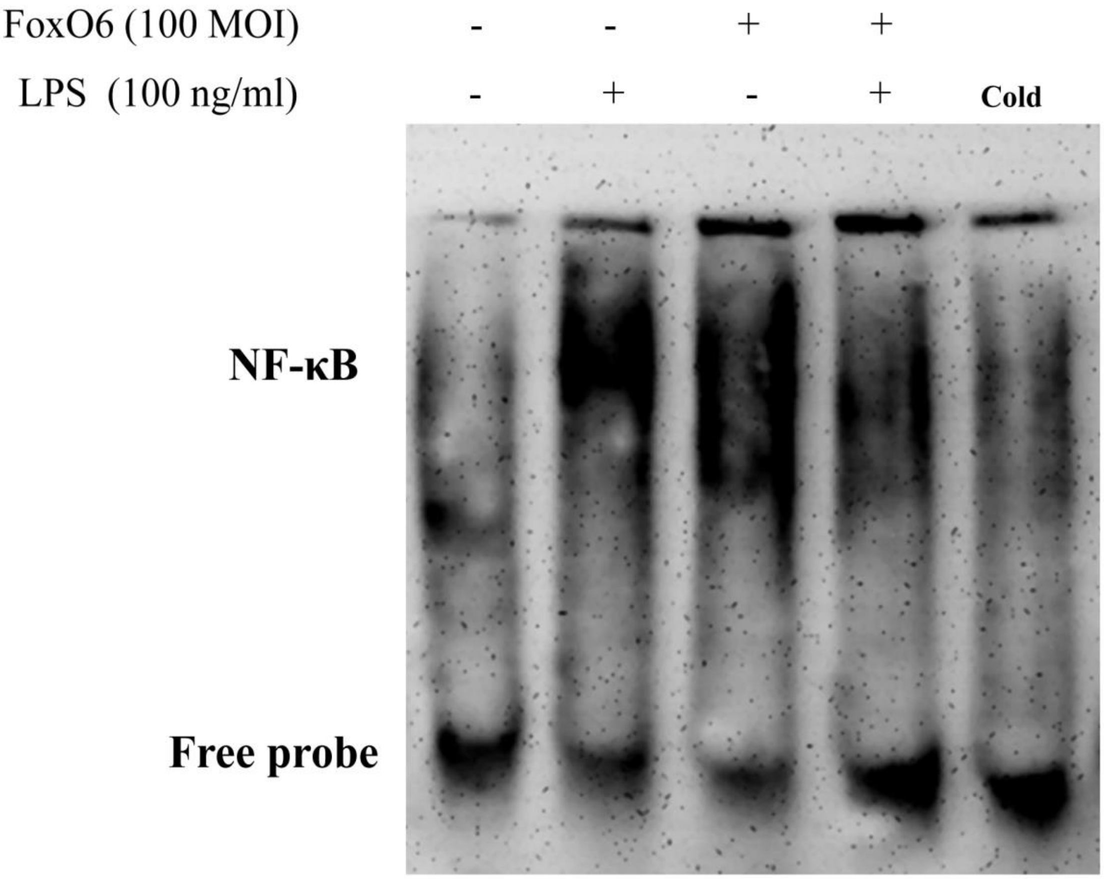

Figure S3. EMSA was used to compare nuclear NF-κB binding activities in HepG2 cells pre-incubated with FoxO6. Results are representative of three independent experiments.

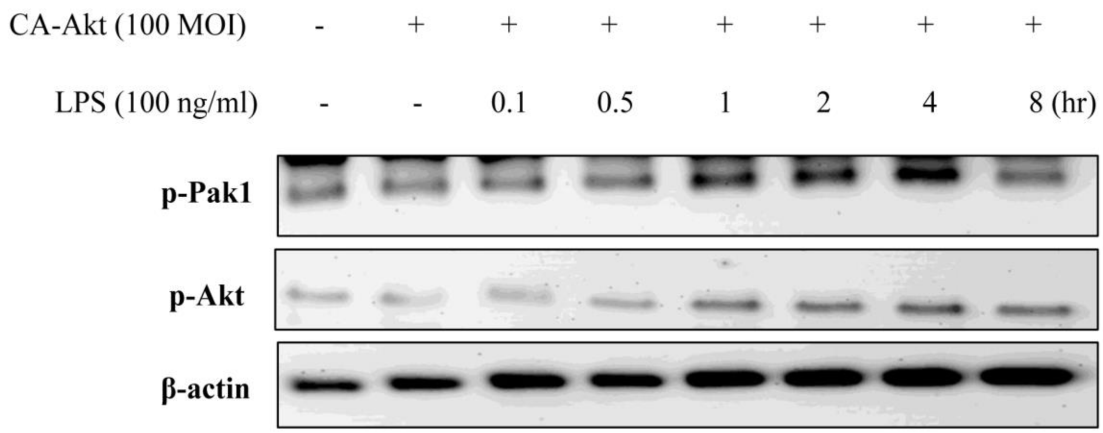

Figure S4. Increases in the phosphorylations of Pak1 and Akt in HepG2 cells exposed to oxidative stress. Cells were grown to 80% confluence in 100 mm dishes in DMEM medium, pre-treated (1 day) with CA-Akt (100 MOI), and then stimulated with 100 ng/ml LPS for 0.1 to 8h in the absence or presence of CA-Akt (100 MOI). Levels of phosphorylated Pak1 and Akt were determined in cell extracts.

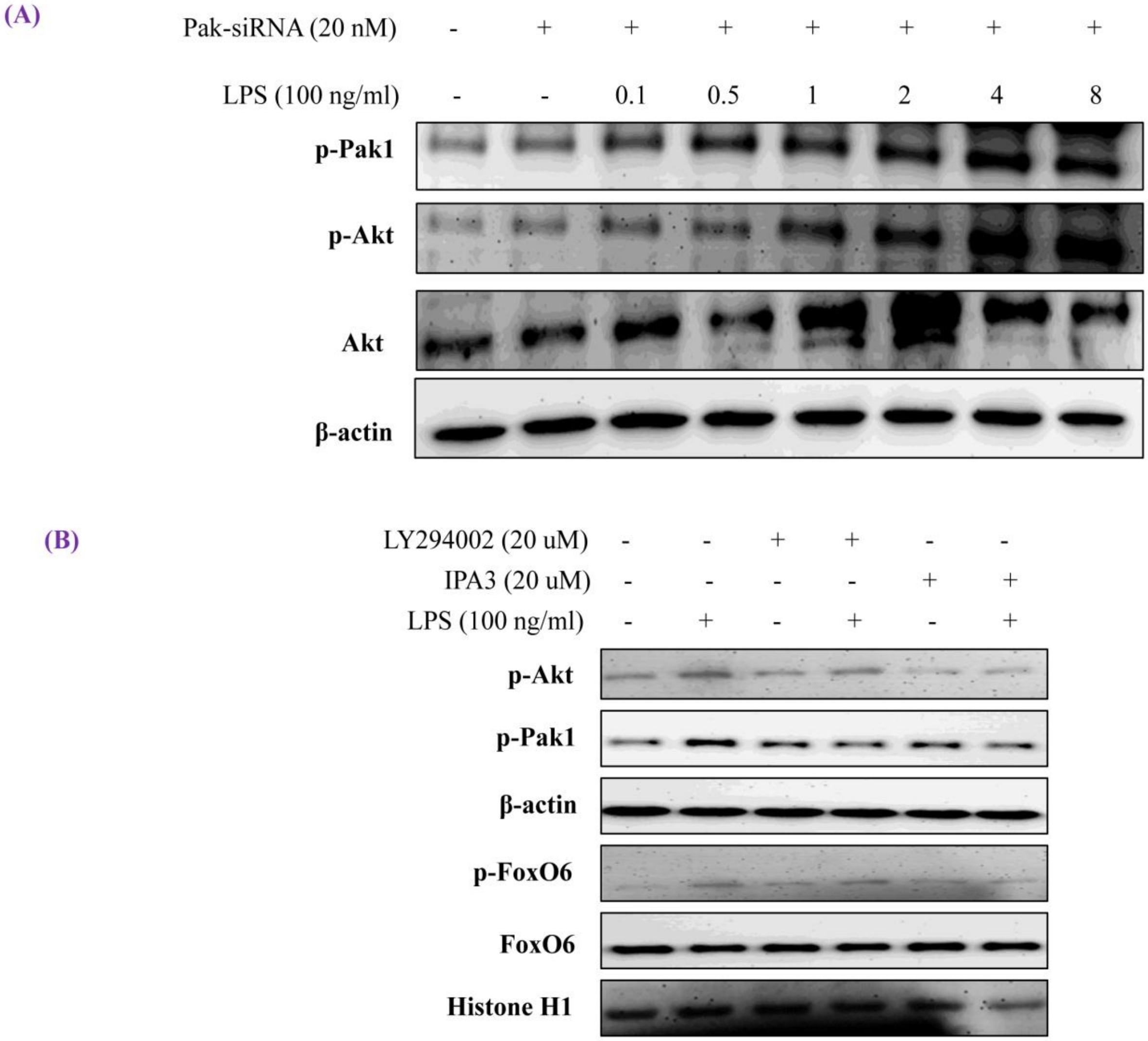

Figure S5. Regulation of FoxO6 by changes in the activities of Pak1 and Akt induced by LPS in HepG2 cells. (A) Cells were transfected (2 days) or not with Pak1-siRNA (20 nM) and then stimulated with 100 ng/ml LPS for 0.1 to 8 hr. Levels of phosphorylated Pak1 and Akt were assessed in cell extracts. (B) Cells pre-treated (1 hr) or not with IPA-3 or LY294002 (20 μM; Pak1 and Akt inhibitors, respectively) and then stimulated with 100 ng/ml LPS for 2 hr. Levels of phosphorylated Pak1, Akt, phosphorylated FoxO6, and total FoxO6 were assessed in cell extracts.
